# Myelomeningocele: neglected aspects

**DOI:** 10.1007/s00467-007-0663-3

**Published:** 2008-08-01

**Authors:** Christopher R. J. Woodhouse

**Affiliations:** Adolescent Urology, University College, 235 Euston Road, London, NW1 2PR UK

**Keywords:** Neurogenic bladder, Myelomeningocele, Long-term outcome, Paediatric nephrology, Education

## Abstract

The commonest cause of neurogenic bladder in children is myelomeningocele. Survival of children is much improved in the Western world, but by 35 years old, about 50% will have died. In adults, the commonest causes of death are lung and heart diseases. All physical aspects deteriorate with age, especially in those with thoracic lesions. Those who walk in childhood have a 20–50% chance of becoming wheelchair dependent as adults. Immobility, poor respiratory reserve, obesity, latex allergy and worsening kyphoscoliosis contribute to the increased risks of surgery. It is essential that safe and manageable urine drainage is established in childhood: the bladder never improves with time, and surgical reconstruction becomes progressively more difficult. Independence in adult life will only be possible with intense preparation in childhood. Children must be allowed to join in with family chores and events. Education, both academic and practical, must be encouraged. Skills such as driving, shopping and birth control must be taught. However, even with the best support, less than 40% will have gainful employment. Children who are continent and have lesions below L2 are likely to have normal sexual function. Sexual activity in adolescents, especially in those with hydrocephalus, is limited (but not absent). However, by adult life, about two thirds will have established a regular partnership. All females and those males who are naturally potent are likely to be fertile. There is a high risk of neural tube defects in their offspring unless the female partner takes prophylactic folic acid for 3 months before pregnancy and for first trimester.

## Introduction

Children who are born with myelomeningocele grow up to become adults–a truth which should be self-evident but often seems to be overlooked by doctors and by some health care systems. In contrast, it is a preoccupation with their parents. Passage to adult life brings with it the difficulties of adolescence that are common to all: the beginning of sexual interest but a continuing deterioration in the body as a whole. Sadly, nothing in spina bifida gets better with age. Preparation for adulthood is frequently neglected in childhood, and the consequences of growing up with spina bifida are poorly researched.

## Survival

**Fig. 1:**
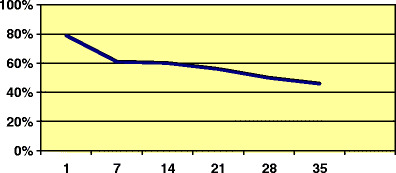
Survival curve for children born with spina bifida

For all babies born with open spina bifida, the prognosis is poor. In underdeveloped countries, congenital anomalies, especially spina bifida, account for nearly 50% of infant deaths, and the rate is inversely correlated with the per capita gross domestic product [1]. In the UK, only 60% of such children have survived into adulthood (Fig. 1) [2]. At all ages, renal failure is the commonest cause of death. In children, the risk of renal failure is strongly related to the sensory level (which may not be the same as the anatomical level in the spine or the level suggested by X-ray). Renal failure is rare with sensory levels at or below L4 and common at or above T10 [3]. However, renal failure can occur even with apparently minor neural tube defects, such as occult spinal dysraphism. In a group of 55 such cases, only 24 patients (43%) had urological symptoms at presentation, and yet all eventually became incontinent, and eight developed renal failure [4].

**Fig. 2:**
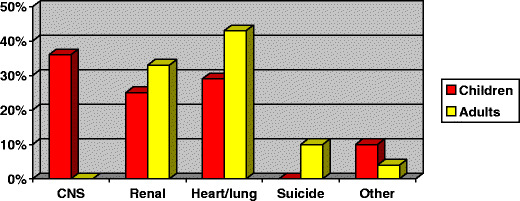
Histogram to show the causes of death in children and adults born with spina bifida. *CNS* Central nervous system

In an unselected series of 695 adults in the UK, 56 were known to have died before their expected time. In the 30 patients whose cause of death could be determined, renal failure accounted for one third. Thirteen died of cardiac or respiratory disease, three committed suicide and one died of cancer (Fig. 2) [5]. There is an increased risk of atherosclerosis in patients with spina bifida, even in the absence of obesity [6].

## Mobility

**Fig. 3:**
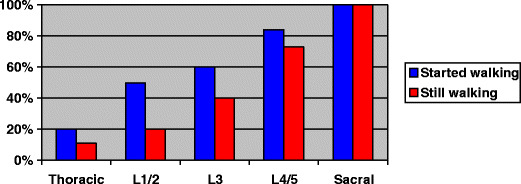
Percentage of children who start ambulation (with or without aids) and percentage who continue to walk up to 9 years 1 month, by spinal level [10]

As the child grows, there is a natural tendency for physical anomalies to deteriorate. The spinal deformity becomes more pronounced. Those who could just walk tend to relapse into a wheelchair. This is illustrated in Fig. 3: those with high lesions, who also have a considerably delayed age of ambulation, have a 20–50% chance of loosing the ability to walk by the age of 10 years. Furthermore, adolescents with spina bifida spend about a half the time in dynamic physical activity as do their normal contemporaries [7].

Unfortunately, just being able to walk does not mean that independence is assured. Even those with sacral lesions who have good muscle strength in the hips may require help with mobility and bowel and bladder care [8]. For those who continue to walk, there may be damage to insensate joints (Charcot arthropathy), which has a prevalence of one in 100 up to the age of 42 years [9]. The percentage of children who start ambulation (with or without aids) and the percentage who continue to walk up to 9 years 1 month, by spinal level, is shown in Fig. 3 [10]. Loss of mobility encourages obesity, which, combined with the collapsed spine, makes much of the lower half of the body invisible to the patient. Attempts to impose weight loss in spina bifida adolescents can lead to eating disorders just as it can in others [11]. Respiratory reserve worsens, making mobility more difficult.

## Surgery in adulthood

The decision to undertake major surgery on an adult with spina bifida should not be taken lightly, and preparation must be meticulous.

If there is to be any significant period of immobility, it may be necessary to have a water bed and nursing by appropriately trained individuals. Even prior to surgery, some patients are limited in the positions in which they can lie; after surgery, the possible positions may be even more limited. There is anecdotal evidence that there is an increased risk of fracture with minimal trauma in patients with spina bifida. Great care must be taken in moving patients in the hospital. Special attention must be given to the position of metal work in the spine, parts of which may come dangerously close to the surface of the skin.

Latex allergy is common in patients with spina bifida, the incidence increasing with age (see below). If there is any suggestion of such a problem, surgery must be performed in a strictly latex-free environment.

With age, respiratory reserve declines. Presumably, increasing kyphoscoliosis is at least a part of the cause. In the author’s own series of adult patients presenting for urinary tract reconstruction, 10% were thought to be unfit for anaesthesia on the basis of lung-function tests [12]. Cardiopulmonary exercise testing (CPX) is rapidly becoming a standard assessment of fitness for major surgery [13]. CPX requires adaptation for use in patients with spina bifida, as it normally is performed on a static bicycle. We are presently investigating the use of upper-limb pedals for CPX testing, especially to measure the anaerobic threshold.

Worsening kyphoscoliosis also makes access to the abdominal cavity more difficult. Patients may be unable to lie flat on the operating table. The distance between the costal margin and the pubis decreases. Rotational deformity of the spine results in a changing relationship between abdomen and chest so that when lying flat on the back at chest level, the abdomen may be rotated to the side. The kidneys may be particularly inaccessible.

**Fig. 4:**
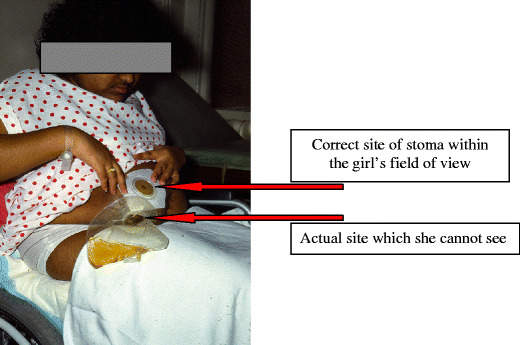
Clinical photograph to illustrate the difficulty that adults with spina bifida have in visualizing the lower half of the abdomen

Neither bowel nor bladder function improves at puberty, though bladder outflow resistance increases (which may bring more danger than advantage). Because of the difficulties with surgery, it is particularly important to establish stable systems in childhood. It should be remembered that the lower half of the abdomen may become invisible to the patient, particularly in women, so that stomata and catheterization sites must be put above the umbilicus (Fig. 4).

## Latex allergy

### Aetiology

Paediatric urologists with a large practice of spina bifida patients are well aware of the problems of latex allergy. Sophisticated strategies have been established to identify patients at risk and to avoid the serious problems of anaphylaxis, especially under anaesthetic. In the United States, the Shriners Myelodysplasia Hospitals are entirely latex free. Adult urologists are much less aware of this potentially fatal problem. Although there is a general recognition that spina bifida children are particularly at risk, the reasons are unclear. It is tempting to think that the increased number of surgical procedures is to blame. Ellsworth et al. found an overall incidence of latex allergy of 60% in 50 spina bifida children. The mean number of operations in positive patients was 9.5 compared with 6.7 in negative children (*P* = 0.03). There was no difference in the age of the children, the number of years that they had been on clean intermittent catheterisation or the number of abdominal operations that they had undergone [14].

However, there are probably other factors to consider. The presence of latex-specific immunoglobulin (Ig)G was found in 25% of a group of European spina bifida patients aged between 2 and 40 years old. Multivariate analysis showed that atopy, especially to pears and kiwi fruit and a history of five or more operations were significantly and independently associated with latex allergy [15]. On the other hand, in a controlled study of the incidence of latex allergy, it was found that that 26% of spina bifida patients had symptomless latex allergy compared with 5% of patients from an ear, nose and throat clinic, even though both groups had had the same number of operations (mean of 1.65 and 1.95, respectively). There were no positive tests in a control group of urological patients who had had no operations [16]. Latex allergy has also been found in 1.1% of patients on regular haemodialysis [17].

### Presentation

About one third of spina bifida patients will have a history of allergic reactions to latex products [14]. The level of awareness of the problem amongst spina bifida children and their families is low, and so the symptoms should be specifically sought. In some cases, the association is obvious, but in others it may be obscure, such as itchy eyes while using washing-up gloves or genital reactions to condoms. In occasional patients, there is acute anaphylaxis under anaesthetic. All patients once identified should be made aware of the risks and should wear a medic-alert bracelet. During medical procedures, latex use should be reduced to a minimum. There are important warning signs that may alert the clinician to the problem in a previously unsuspected case. In a group of five children (from 17) who were undergoing transurethral bladder stimulation and urodynamics, all had coughing or sneezing for several minutes before bronchospasm and generalised allergic reactions developed [18].

### Management

The most important part of management is identification of patients at risk and prophylaxis. As the incidence is so high in spina bifida patients, a specific enquiry should be made for symptoms in all patients. Minor reactions can be treated with bronchodilators, antihistamines and, occasionally, steroids. It should be remembered that minor reactions may be the forerunner of major anaphylaxis. All who have a suggestive history or unexplained hypotensive episodes under anaesthetic should undergo skin testing and measurement of latex-specific IgE. A latex-free medical environment should be created for patients found to be at risk. In all of four patients on the author’s service who presented with anaphylaxis under anaesthetic, further operations have been uneventful in a latex-free operating theatre (unpublished data).

## Preparation for adulthood

Childhood is a preparation for adulthood. This is true of children with spina bifida as it is for those who are more normal. It should come as no surprise to find that if those with spina bifida are inadequately prepared, they will not become independent. As with everything else in this condition, it is very hard to do. Expectations must be appropriate and special needs met, but without such work in childhood, dependence will continue into adult life. Few countries have adequate arrangements for young dependent adults, and much of the burden will, therefore, continue to fall on the family. It is seldom appropriate to wait for symptoms to “improve with age”. Anything that is not sorted out in childhood will be more difficult to manage in adulthood.

Autonomy of the disabled can be considerably improved by an active but realistic programme of education [19]. Parents who have a positive and hopeful attitude are able to improve their adolescent’s quality of life by about 25% over that which would be predicted for the disability at birth [20]. Unfortunately, even with the highest levels of expectation and ambition, participation in the full range of adolescent activities or household chores is uncommon [21]. Although incontinence is not always a barrier to a fulfilled life, those who are continent have an improved view of self worth. In girls, social acceptance and views of global self-worth and in boys, scholastic competence, social acceptance, physical appearance and behaviour are all improved by continence [22].

In addition, throughout childhood, a whole set of emotional and social problems emerge, though they cannot totally be separated from the physical. Patients become sexually interested without necessarily having the means to indulge or control their desires. Maturity brings new interests. For those who are reasonably intelligent, there is a desire to have normal friends, to go out to the cinema or club and to socialise with a peer group. Many patients are able to work, some in very demanding areas including medicine, though special training or work facilities may be needed. For the mentally retarded, appropriately stimulating occupations must be found. All of this requires help. At the very least, there has to be an infrastructure of sympathetic friends who will take a wheelchair-bound person to places that often are not too well equipped to accommodate them. Social research shows that buildings and transport systems are poorly adapted to the needs of the handicapped.

All children who are born with congenital anomalies have a desire to be normal and to be treated as such. It is essential to encourage children with spina bifida to look after themselves and to take part in normal family life right from the start.

## Sexual development

Sexual development of handicapped patients has been the subject of little research. The most obvious public attention arises when there is a moral or ethical problem. For example, a difficult area of community policy is how to prevent unwanted pregnancies and sexual abuse in affected girls of low intelligence. Those with severe congenital disabilities often fail to develop normal sexuality because of lack of privacy and dependence on others for normal daily living. They have low social and sexual confidence. Surprisingly, however, even those who can walk and whose spina bifida is “hidden” have major sexual problems. They will have uncertain bladder and bowel control, which leads to an unwillingness to mix with their peers on an equal basis. It is most important not to imagine that an apparently minor level of neurological disability means that sexuality is normal [23]. The physical aspects of sexual function that depend on the brain are generally intact, whereas those that depend on the spinal cord will be damaged in line with the neurological level.

When children are brought up in the mainstream of education and integrated in school, the social results are excellent. In a study to compare 11 dimensions of self-image in adolescents with spina bifida with those of their peers, there was no difference in ten. Unfortunately, the 11th was the dimension of sexuality, which was significantly below normal, especially in females [24]. Spina bifida adolescents are often ignorant of even very straightforward aspects of sexuality, which should be taught within the family. The ordinary facts of reproductive life often are not given. Up to 23% of girls do not know about the hygienic management of menstruation [25, 26]. It is not surprising, therefore, that adolescents have very little sexual contact.

In females, as in males, sexual function, as defined by sexual sensation and orgasm, is dependent on neurological level. Most with levels below L2 are thought to have normal sexual sensations, as are most with urinary continence. Only about 20% of those with higher levels or with urinary incontinence have normal sexual function [27]. Some women appear to have powerful contractions of the detrusor in response to sexual stimulation or orgasm. The contractions are painful if the bladder is empty and cause incontinence if any urine is present. They are not abolished by clam cystoplasty, but they are if the bladder is removed and a substitution cystoplasty performed (unpublished data).

**Fig. 5:**
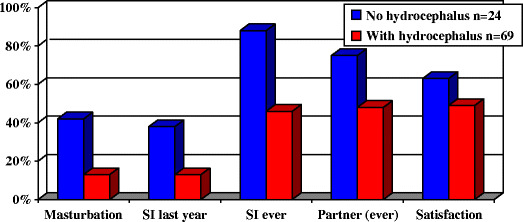
Histogram showing sexual activity in women with spina bifida [28]. *SI* Sexual intercourse

A recent study from the Netherlands has shown that, in practice, sexual activity is less common than might have been suspected, especially in those with hydrocephalus (Fig. 5) [28]. Erection and ejaculation in males is largely under neurological control. All males with intact sacral reflexes and urinary continence are potent. With absent sacral reflexes, 64% with levels below D10 and 14% with levels above D10 are potent. There is doubt about the true sexual nature of such erections [27].

**Fig. 6:**
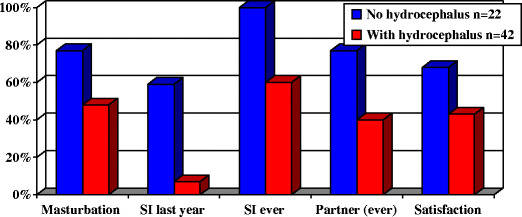
Histogram showing sexual activity in men with spina bifida [28]. *SI* Sexual intercourse

Again, male patients in a Dutch study had less than expected sexual activity, especially if they had hydrocephalus (Fig. 6) [28]. Impotence responds to conventional management such as intracorporeal injection [29]. Sildenafil may be used with appropriate dose reduction. In the only trial to date in this group, dose escalation was used with patients as their own controls. Eighty percent of men responded to a dose of 50 mg. Although one patient subsequently responded to 100 mg, it was recommended that such a high dose should not be used in spina bifida. Five of the 11 responders in the series were wheelchair bound [30, 31]. In view of the possibility that impotence is associated with azoospermia, the prospects for fertility may not be improved.

## Fertility

Fertility in females is thought to be normal. The main problem is, of course, the risk of a neural tube defect in the offspring, which is discussed below. Folic acid prophylaxis considerably reduces the risk. A secondary cause is the obesity commonly seen in spina bifida adolescents. Unfortunately, the incidence of neural tube defect pregnancies is nearly double in women who are obese at the time of conception (body mass index >29 kg/m^2^) [32].

There have been several reports of favourable pregnancy outcomes in women with spina bifida, though one group summarised their experiences with difficult pregnancies in (medically) difficult patients from difficult (socially deprived) families [33]. Several specific problems have been identified. Urinary tract infections are almost invariable; bladder function and mobility often deteriorate, though not permanently; the deformed and, often, small pelvis makes accommodation of the foetus difficult, leading to premature labour and an increased need for caesareans [33, 34]. In a series of 20 pregnancies, 35% were delivered before 37 weeks. Eight required a caesarean section, all for obstetric indications, most commonly disproportion [33]. Four of five wheelchair-bound women required caesarean section compared with eight of 18 walkers [35].

For females in general and spina bifida girls in particular, one of the great medical success stories of the last 25 years has been the discovery of the prophylactic role of folic acid. In a double-blind placebo-controlled trial in 1,195 women with high-risk pregnancy (previous birth of a child with neural tube defect or affected parent), there were six affected foetuses in the treated group versus 21 in the untreated group [36]. If the father has spina bifida, the pregnancy is also at risk.

**Fig. 7:**
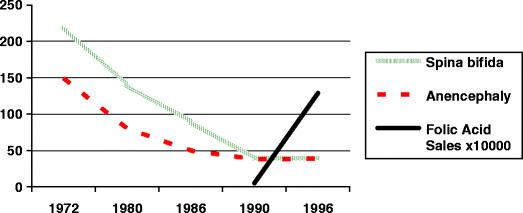
Graph showing the incidence of births of children with neural tube defects by year. The* black line* shows the sales of folic acid

The incidence of all neural tube defects in babies in the Western world has diminished in the last 30 years. Even before the discovery of the protective effects of folic acid, the overall incidence had been falling. In a recent study from the United Kingdom, it was shown that the incidence of neural tube defects started to fall about 18 years before the use of folic acid began to rise. The incidence fell from about 225/100,000 live births to about 48/100,000 live births between 1972 and 1990. The number of sales of folic acid was less than 100,000 per year until 1990, rising to 1.2 million per year by 1996 (Fig. 7) [37]. However, the main protection against the conception of a baby with a neural tube defect is to give the mother folic acid supplements in the 3 months before conception and for the first trimester [36, 38]. The Department of Health in the United Kingdom now recommends that women who are at high risk of conceiving a baby with a neural tube defect should take 5 mg per day whereas 0.8 mg per day is sufficient for other women [39]. Despite this prophylaxis, there remains a small risk of an affected pregnancy. At least one cause appears to be an in-born error of folic acid metabolism, which was found in 16 women who gave birth to two successive babies with myelomeningocele in spite of prophylaxis [40].

The use of selective pregnancy termination in cases of neural tube defect is as much a social as a medical issue. In a series in South Australia between 1966 and 1991, the prevalence of affected pregnancies did not change, but the number of affected live births fell by 84% from 2.29 to 0.35/1,000 with an active programme of prenatal diagnosis and termination [41].

In males infertility appears to be a common problem. In those with higher lesions, it might be thought that it would due to impotence. Although this is undoubtedly true in part, preliminary results of a continuing study have identified another problem. In ten impotent males with spina bifida, all were found to be azoospermic on analysis of semen obtained by electroejaculation. On testicular biopsy, all had Sertoli cells only [42]. Poor semen quality has also been reported in men with acquired spinal lesions using electroejaculation, especially if ejaculation is infrequent [23].

No figures are available for the overall incidence of infertility in males. However, it is interesting to note a study of 49 adults with spina bifida in South Wales (UK): 16 men and 15 women were married or had a regular partner. Twenty-eight (90%) of these married couples had children. Success in partnership and fertility were said to be unrelated to continence or mobility [43].

## Independence and work

Few data are available on levels of independence and employment of adults with spina bifida. The greater the handicap, both physical and mental, the less likely is a successful outcome. Even so, many are able to occupy themselves with work at home or in protected environments, with or without pay. The social outcome in an unselected group of adults is shown in Table 1 [44].

**Table 1:** Social outcomes in adults with spina bifida

	Below L3	L3-T11	Above T11
	*n* = 24	*n* = 15	*n* = 12
IQ> 80	21	11	6
Walker	16	0	0
Independent	14	5	2
Driver	14	4	2
Employed	9	4	2

In a Swedish study, 38% of young adults with spina bifida were in gainful employment (compared with 47% with traumatic paraplegia). Not surprisingly, a better outcome was found in walkers with higher educational achievements [45].

## Conclusion

The management of children with spina bifida has improved considerably in the last 50 years. In particular, understanding of the neuropathic bladder has lead to less urinary infection, less renal damage and better continence. We now need to establish how aggressive it is necessary to be in childhood to produce a good bladder in adults. Sadly, inadequate attention has been paid to preparation for independent adult life for these individuals. Families and support agencies must remember that childhood is only about 25% of the human lifespan. It must be used, as it is with normal children, to prepare for the remaining 75%.

## Questions

Multiple choice questions (answers appear following reference list)

1. On present data, what percentage of babies born with myelomeningocele have survived into adult life?
  - 75–80%
  - 65–70%
  - 60–64%
  - 40–45%
2. In adults born with myelomeningocele, what is the commonest cause of death?
  - Renal failure
  - Suicide
  - Infected ventriculoperitoneal shunt
  - Respiratory failure
3. In wheelchair-bound males born with myelomeningocele, which of the following may improve with puberty?
  - Bowel control
  - Manual dexterity
  - Respiratory reserve
  - Bladder outflow resistance
4. In which men with myelomeningocele are erections most likely to be normal?
  - Sensory level below D10
  - Absent history of hydrocephalus
  - Normal serum testosterone
  - In-tact sacral reflexes
5. Which of the following is the most important prophylaxis to reduce the risk of neural tube defects in babies conceived by women born with myelomeningocele?
  - Folic acid 5 mg daily in the first trimester
  - Folic acid 5 mg daily in the first two trimesters
  - Folic acid 5 mg daily for at least 3 months before conception and in the first trimester
  - Folic acid 5 mg daily for at least 3 months before conception
